# Malnutrition and the Post-Acute Sequelae of Severe Acute Respiratory Syndrome Coronavirus 2 Infection: A Multi-Institutional Population-Based Propensity Score-Matched Analysis

**DOI:** 10.3390/life14060746

**Published:** 2024-06-12

**Authors:** Cheng-Ya Lee, Yung-Chun Liang, Wan-Hsuan Hsu, Ya-Wen Tsai, Ting-Hui Liu, Po-Yu Huang, Min-Hsiang Chuang, Kuo-Chuan Hung, Mei-Chuan Lee, Tsung Yu, Chih-Cheng Lai, Tzu-Chieh Weng, Jheng-Yan Wu

**Affiliations:** 1 Division of Cardiovascular Surgery, Department of Surgery, Chi Mei Medical Center, Tainan 710, Taiwan; lee19920311@hotmail.com.tw; 2 Department of Internal Medicine, Chi Mei Medical Center, Tainan 710, Taiwan; paul2760952@gmail.com (Y.-C.L.); nicolar930@gmail.com (W.-H.H.); b0807246@gmail.com (P.-Y.H.); 3 Division of Preventive Medicine, Chi Mei Medical Center, Tainan 710, Taiwan; rositatsai@gmail.com; 4 Department of Psychiatry, Chi Mei Medical Center, Tainan 710, Taiwan; liu.tingkle@gmail.com; 5 Division of Nephrology, Department of Internal Medicine, Chi Mei Medical Center, Tainan 710, Taiwan; 60028a@gmail.com; 6 Department of Anesthesiology, Chi Mei Medical Center, Tainan 710, Taiwan; ed102605@gmail.com; 7 School of Medicine, College of Medicine, National Sun Yat-sen University, Kaohsiung City 804, Taiwan; dtmed141@gmail.com; 8 Department of Public Health, College of Medicine, National Cheng Kung University, Tainan 701, Taiwan; pharmacistmeichuanlee@gmail.com (M.-C.L.); tsung.yu.ncku@gmail.com (T.Y.); 9 Department of Pharmacy, Chi Mei Medical Center, Tainan 710, Taiwan; 10 Department of Intensive Care Medicine, Chi Mei Medical Center, Tainan 710, Taiwan; 11 Division of Hospital Medicine, Department of Internal Medicine, Chi Mei Medical Center, Tainan 710, Taiwan; 12 Department of Nutrition, Chi Mei Medical Center, Tainan 710, Taiwan

**Keywords:** malnutrition, nutrition deficiency, undernutrition, COVID-19, mortality

## Abstract

Coronavirus disease 2019 (COVID-19), caused by severe acute respiratory syndrome coronavirus 2 (SARS-CoV-2), has led to a global health crisis, exacerbating issues like malnutrition due to increased metabolic demands and reduced intake during illness. Malnutrition, a significant risk factor, is linked to worse outcomes in patients with COVID-19, such as increased mortality and extended hospital stays. This retrospective cohort study investigated the relationship between malnutrition and clinical outcomes within 90–180 days using data obtained from the TriNetX database. Patients aged >18 years diagnosed with COVID-19 between 1 January 2022, and 31 March 2024 were enrolled in the study. The propensity score-matching (PSM) method was used to match patients with malnutrition (malnutrition group) and those without malnutrition (control group). The primary composite outcome was the cumulative hazard ratio (HR) for post-COVID-19 condition, all-cause hospitalization, and all-cause mortality between 90 days and 180 days after COVID-19 diagnosis. The secondary outcomes were the individual components of the primary outcomes. Two cohorts, each consisting of 15,004 patients with balanced baseline characteristics, were identified using PSM. During the 90–180-day follow-up period, the malnutrition group exhibited a higher incidence of all-cause hospitalization, mortality, or post-COVID-19 condition (HR = 2.315, 95% confidence interval: 2.170–2.471, *p* < 0.0001). Compared with patients with COVID-19 without malnutrition, those with malnutrition may be associated with a higher risk of adverse clinical outcomes.

## 1. Introduction

Coronavirus disease (COVID-19), an acute respiratory syndrome caused by severe acute respiratory syndrome coronavirus 2 (SARS-CoV-2), has triggered a global pandemic and public health outbreak [1]. As of 5 May 2024, over 700 million cases and 7 million deaths have been reported worldwide [2]. The spectrum of symptoms in infected individuals varies widely; some patients remain asymptomatic, while others develop mild symptoms such as cough, chills, fever, fatigue, and dyspnea [3,4]. More severe manifestations include sepsis, acute respiratory distress syndrome, heart failure, septic shock, and multi-organ dysfunction due to the acute inflammatory response [5,6]. Beyond these immediate effects, approximately 10% of individuals experience long-term consequences known as the post-acute sequelae of SARS-CoV-2 infection (PASC) or “long COVID.” PASC can involve a range of persistent symptoms, including chronic fatigue, respiratory difficulties, cognitive impairment, and cardiovascular issues, extending for months beyond the initial recovery period [7,8,9].

Building on an understanding of the impact of COVID-19, the factors that can exacerbate the severity of the disease must be investigated. Malnutrition, which can manifest in acute, subacute, or chronic form, is one such factor. It is characterized by progressive weight loss, insufficient energy intake, muscle and fat loss, fluid accumulation, and reduced grip strength [10,11]. It is a significant independent risk factor for increased morbidity and mortality across various diseases, as it leads to heightened susceptibility to infections or superinfections [12].

Malnutrition may influence the severity of COVID-19 through several mechanisms, including impairment of the immune response, increased inflammation, and delayed recovery. Deficiencies in key nutrients, such as vitamins A, C, and D, zinc, and protein–energy malnutrition can reduce lymphocyte proliferation, impair immune function and antibody production, and weaken the mucosal barriers that are crucial for preventing pathogen entry. These deficiencies can compromise pulmonary function, which can weaken the respiratory muscles and reduce the pulmonary immune defenses, thereby facilitating more severe respiratory symptoms and extensive viral damage [10,11,12].

Previous meta-analyses have indicated that nearly 49% of patients with COVID-19 experience malnutrition [13]. Observational studies have suggested that malnutrition in these patients correlates with poorer outcomes, including increased rates of mechanical ventilation, mortality, and prolonged hospital stay [14,15,16,17,18,19].

However, much of the existing evidence is derived from retrospective studies conducted at a single center. Therefore, more comprehensive studies with larger sample sizes are needed. This study aimed to explore the association between malnutrition and clinical outcomes in patients with SARS-CoV-2 infection over a period of 90–180 days using an international database.

## 2. Materials and Methods

### 2.1. Data Source

The data used in this study were collected from the TriNetX Research Network. The TriNetX database shares electronic medical record data (diagnoses, procedures, medications, laboratory values, genomic information, and types of visits) of approximately 140 million individuals at 119 healthcare organizations (HCOs) [20]. Numerous observational studies have utilized the TriNetX database, particularly when conducting COVID-19 research [21,22,23,24]. The TriNetX platform provides integrated tools for analyzing patient-level data and presents outcomes to researchers as consolidated reports. Considering that the data utilized from TriNetX were anonymized, written informed consent was not required. This study was approved by the Institutional Review Board of the Chi Mei Medical Center (approval no. 11302-E01).

### 2.2. Patient Selection and Exposure

**Figure 1:**
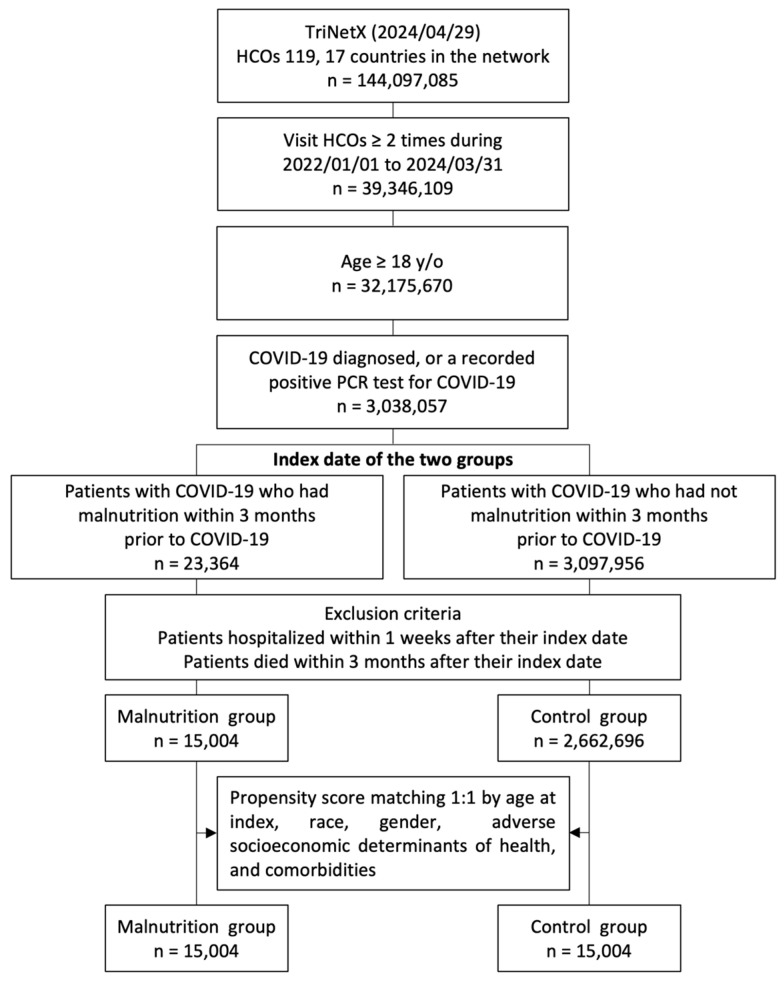
Flowchart of the participant selection process. HCOs: healthcare organizations; y/o: years old; PCR: polymerase chain reaction.

Patients aged >18 years who had more than two visits to HCOs from 1 January 2022 to 31 March 2024 and were diagnosed with COVID-19 (confirmed by a positive polymerase chain reaction test [laboratory test code with TNX: LAB:9088] or an International Classification of Diseases, Tenth Revision, Clinical Modification (ICD-10-CM) code U07.1) [25,26,27] were included in the study. To ensure consistency in terms of disease severity, patients who died within 90 days or were hospitalized within 7 days after contracting COVID-19 were excluded. Patients were further divided into the malnutrition and control groups based on whether they exhibited signs of malnutrition (ICD-10 cm codes: E40–E46) within 90 days before the index date. Our initial cohort from 1 January 2022 to 31 March 2024, consisted of 3,038,057 patients with COVID-19, including 23,364 with malnutrition and 3,014,693 without malnutrition (Figure 1).

### 2.3. Covariates

To balance the distribution between groups at baseline, we selected covariates for matching based on CDC [28]. The baseline variables used to match the two study groups included (a) demographics such as age, sex, and race; (b) potential health risks linked to socioeconomic factors, including housing and financial conditions, educational attainment and literacy levels, employment status, and occupational exposure to hazards; and (c) comorbid conditions such as alcohol-related disorders, nicotine dependence, hypertension, hyperlipidemia, diabetes mellitus, neoplasms, chronic diseases of the lower respiratory tract, liver diseases, chronic kidney disease, end-stage renal disease, cerebrovascular disease, heart failure, and atrial fibrillation.

### 2.4. Outcomes

The primary outcome of this study was a composite of all-cause hospitalization, all-cause mortality, or post-COVID-19 condition. The secondary outcomes were the individual components of the primary outcome, including all-cause hospitalization, all-cause mortality, and post-COVID-19 condition. The follow-up period was 90–180 days after the index date.

### 2.5. Statistical Analysis

Statistical analyses were performed using the integrated functions of the TriNetX platform. The baseline characteristics of the malnutrition and control groups were expressed as the means, standard deviations, counts, and percentages. To correct imbalances in baseline covariates, a 1:1 PSM was employed. The PSM technique utilized a nearest-neighbor matching algorithm with a caliper width set at 0.1 of pooled standard deviations. Variables with a standardized difference of less than 0.1 between groups were considered adequately matched. Following PSM, the cumulative incidence of each outcome was estimated using Cox regression models and the results were expressed as hazard ratios (HRs) with 95% confidence intervals (CIs). Additionally, Kaplan–Meier curves were generated to assess the survival distributions between the groups, with significance evaluated using the log-rank test. A *p* value of <0.05 was considered significant.

### 2.6. Subgroup Analysis

Subgroup analyses were performed using a stratified approach to explore variations in outcomes based on sex, age, race, vaccination status, and the antiviral agent used.

## 3. Results

### 3.1. Demographic Characteristics

The baseline characteristics of the two groups are presented in Table 1. Prior to PSM, notable differences were noted in the demographic and health profiles of the two groups. Specifically, individuals in the malnutrition group were generally older than those in the control group (mean age: 66.0 ± 17.5 vs. 50.6 ± 19.2). The malnutrition group also had a higher proportion of women (58.7% vs. 46.4%). In terms of comorbidities, the malnutrition group exhibited a higher prevalence of alcohol-related disorders (8.9% vs. 2.6%), nicotine dependence (17.7% vs. 7.9%), hypertension (52.4% vs. 30%), hyperlipidemia (41.4% vs. 28.4%), diabetes mellitus (29.4% vs. 14.4%), neoplasms (34.3% vs. 20.5%), chronic lower respiratory diseases (25.5% vs. 15.3%), liver diseases (15.9% vs. 6.3%), chronic kidney disease (22.4% vs. 6.6%), end-stage renal disease (4.8% vs. 1.1%), cerebrovascular diseases (20.1% vs. 4.9%), atrial fibrillation and flutter (16.5% vs. 4.9%), and ischemic heart diseases (27.3% vs. 9.5%). Following PSM, both groups comprised 15,004 patients, each with well-balanced demographic characteristics, as summarized in Table 1.

### 3.2. Primary Outcomes

**Figure 2:**
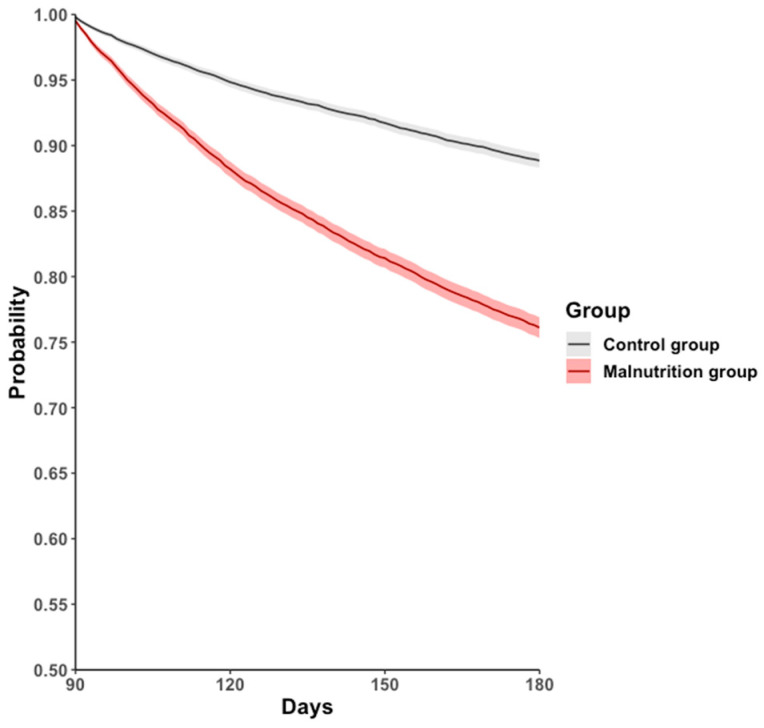
Kaplan–Meier event-free curves for the primary outcome: a composite of all-cause hospitalization, all-cause mortality, or post-COVID-19 condition.

During the 90–180-day follow-up period, the all-cause hospitalization, mortality, or long COVID-19 rate was higher in the malnutrition group (17.9%) than in the control group (9.2%), with an HR of 2.315 (95% CI: 2.170–2.471, *p* < 0.001; Figure 2, Table 2).

### 3.3. Secondary Outcomes

For the individual component outcomes during the 90–180-day follow-up period, the malnutrition group showed a higher risk of all-cause hospitalization (HR = 2.151, 95% CI: 2.008–2.304), all-cause mortality (HR = 4.459, 95% CI: 3.699–5.376), and post-COVID-19 condition (HR = 2.707, 95% CI: 1.913–3.832) than the control group (Table 2).

### 3.4. Subgroup Analysis

The risk of the primary outcome was examined based on age, sex, race, vaccination status, and the antiviral agent used (Table 3). Subgroup analyses showed consistent results for each subgroup. In terms of the other secondary outcomes, similar trends were observed in the subgroup analyses (Table 4).

## 4. Discussion

This study used a large sample size to investigate the association between malnutrition and clinical outcomes in patients with COVID-19 over a 90–180-day follow-up period. Our findings highlight that patients with COVID-19 experiencing malnutrition have a significantly higher risk of severe outcomes, including all-cause hospitalization, all-cause mortality, and post-COVID-19 condition. Notably, the effect size of all-cause mortality in the malnutrition group was fourfold higher than that in the control group, indicating a substantial impact of nutritional status on COVID-19 severity.

The underlying mechanisms by which malnutrition exacerbates COVID-19 severity involve both direct and indirect effects on patient health. Malnutrition weakens immune defense mechanisms, as evidenced by a reduced lymphocyte count and impaired phagocytic function, which are critical for combating viral infections [29,30]. Deficiencies in essential proteins, vitamins, and minerals further weaken physical barriers and cellular immunity. This predisposition not only increases susceptibility to severe infections but also increases the risk of prolonged illness and complications [31,32,33,34].

Furthermore, malnutrition exacerbates the inflammatory cascade associated with COVID-19. It induces an imbalanced cytokine profile, promoting inflammatory pathways that can lead to the occurrence of cytokine storm syndrome, a known predictor of severe outcomes in COVID-19. Uncontrolled inflammation can result in tissue damage, multi-organ failure, and heightened mortality risk [32,35,36,37,38,39].

Consistent with previous studies, malnutrition amplifies the severity of infectious diseases, including COVID-19 [3,12,40,41,42,43,44]. A meta-analysis of 12 studies demonstrated a similar trend, showing that malnourished patients had significantly higher odds of in-hospital mortality (odds ratio = 3.43, 95% CI: 2.55–4.60) during a 90-day follow-up [45]. Our study builds on these findings by including non-hospitalized patients and extending the follow-up duration, thus providing a more comprehensive view of the impact of malnutrition on COVID-19 outcomes in various settings. Additionally, our study addressed some of the limitations observed in previous studies, such as small sample sizes and short follow-up periods, by utilizing a larger and more diverse cohort with a longer observation period. This approach enhances the generalizability and applicability of our findings, suggesting robust associations across various healthcare settings and populations.

The strong association between malnutrition and adverse COVID-19 outcomes underscores the necessity of early nutritional screening and intervention in patients with COVID-19. The implementation of nutritional support strategies can potentially reduce disease severity and improve clinical outcomes. Physicians and dietitians should consider integrated care pathways that incorporate nutritional assessments and tailored interventions as part of standard care for patients with COVID-19.

Although our findings are significant, they highlight the need for further research to explore the causal relationships and effectiveness of specific nutritional interventions in improving COVID-19 prognosis. Prospective and randomized controlled trials are essential to determine the specific nutrients and dietary interventions that are most effective in mitigating the impact of malnutrition on COVID-19 severity.

This study has several strengths. First, although the existing research often relies on data from a single hospital with limited sample sizes and generalizability, our study draws from a vast and diverse population. This diversity enhances the relevance and generalizability of our findings to broader real-world settings. Second, we employed PSM to ensure comparability between the groups, effectively controlling for potential confounding factors related to the variables of interest and observed outcomes. Finally, the consistency observed across various subgroup analyses adds further credibility to our findings.

This study has some limitations. First, potential information bias and coding errors in the electronic health records database may have occurred; however, these errors were likely consistent between the groups, which minimizes their impact on our findings [46]. Second, relying on ICD-10 cm codes to determine the history of malnutrition may underestimate its true prevalence. Third, although our data suggest significant associations, they do not establish causality. Fourth, due to database limitations, we could not determine the countries from which these patients originated. Finally, to control for disease severity and reduce heterogeneity, we excluded patients who were initially hospitalized and deceased within 90 days from the index date, limiting the generalizability of our findings.

## 5. Conclusions

This study demonstrated that malnutrition is associated with a significantly higher risk of all-cause hospitalization, mortality, or post-COVID-19 condition compared with the control group. Therefore, malnutrition is a potential risk factor for poor clinical outcomes in patients with COVID-19. These findings highlight the critical role of malnutrition in exacerbating the severity of COVID-19. The robustness of the data, enhanced by the large sample size and diverse patient population, provides compelling evidence supporting the integration of nutritional evaluations and interventions in the management of COVID-19.

## Figures and Tables

**Table 1:** Baseline characteristics of the study participants before and after the implementation of propensity-score matching.

Variables	Before Matching			After Matching		
	Malnutrition Group (n = 15,004)	Control Group (n = 2,662,662)	Std Diff	Malnutrition Group (n = 15,004)	Control Group (n = 15,004)	Std Diff
Age at index, years						
Mean ± SD	66.0 ± 17.5	50.6 ± 19.2	0.84	66.0 ± 17.5	66.8 ± 17.3	0.05
Gender, n (%)						
Female	6960 (46.4)	1,562,584 (58.7)	0.25	6960 (46.4)	7027 (46.8)	<0.01
Male	7208 (48)	992,649 (37.3)	0.22	7208 (48)	7125 (47.5)	0.01
Race, n (%)						
White	8687 (57.9)	1,557,689 (58.5)	0.01	8687 (57.9)	8695 (58)	<0.01
American Indian or Alaska Native	47 (0.3)	7481 (0.3)	0.01	47 (0.3)	41 (0.3)	<0.01
Native Hawaiian or Other Pacific Islander	32 (0.2)	12,099 (0.5)	0.04	32 (0.2)	37 (0.2)	<0.01
Black or African American	2373 (15.8)	370,888 (13.9)	0.05	2373 (15.8)	2360 (15.7)	<0.01
Asian	863 (5.8)	111,754 (4.2)	0.07	863 (5.8)	924 (6.2)	0.02
Other Race	608 (4.1)	116,846 (4.4)	0.02	608 (4.1)	655 (4.4)	0.02
Social determinants of health associated with adverse outcomes, n (%)						
Housing and economic circumstances	584 (3.9)	55,768 (2.1)	0.11	584 (3.9)	533 (3.6)	0.02
Employment and unemployment	224 (1.5)	17,091 (0.6)	0.08	224 (1.5)	183 (1.2)	0.02
Education and literacy	83 (0.6)	5473 (0.2)	0.06	83 (0.6)	62 (0.4)	0.02
Occupational exposure to risk factors	23 (0.2)	5176 (0.2)	0.01	23 (0.2)	24 (0.2)	<0.01
Comorbidities, n (%)						
Alcohol-related disorders	1339 (8.9)	69,496 (2.6)	0.27	1339 (8.9)	1175 (7.8)	0.04
Nicotine dependence	2660 (17.7)	209,470 (7.9)	0.30	2660 (17.7)	2530 (16.9)	0.02
Hypertension	7865 (52.4)	799,286 (30)	0.47	7865 (52.4)	8061 (53.7)	0.03
Hyperlipidemia	6174 (41.1)	754,990 (28.4)	0.27	6174 (41.1)	6242 (41.6)	<0.01
Diabetes mellitus	4407 (29.4)	382,180 (14.4)	0.37	4407 (29.4)	4461 (29.7)	<0.01
Neoplasms	5143 (34.3)	546,655 (20.5)	0.31	5143 (34.3)	5146 (34.3)	<0.01
Chronic lower respiratory diseases	3776 (25.2)	408,114 (15.3)	0.25	3776 (25.2)	3800 (25.3)	<0.01
Diseases of liver	2390 (15.9)	166,690 (6.3)	0.31	2390 (15.9)	2307 (15.4)	0.02
Chronic kidney disease	3358 (22.4)	176,135 (6.6)	0.46	3358 (22.4)	3278 (21.8)	0.01
End-stage renal disease	724 (4.8)	30,343 (1.1)	0.22	724 (4.8)	641 (4.3)	0.03
Cerebrovascular diseases	3010 (20.1)	151,654 (5.7)	0.44	3010 (20.1)	2917 (19.4)	0.02
Heart failure	3015 (20.1)	129,363 (4.9)	0.47	3015 (20.1)	2820 (18.8)	0.03
Atrial fibrillation and flutter	2474 (16.5)	129,184 (4.9)	0.38	2474 (16.5)	2417 (16.1)	0.01
Ischemic heart diseases	4093 (27.3)	253,795 (9.5)	0.47	4093 (27.3)	4024 (26.8)	0.01

Std Diff: standardized difference. A standardized difference (Std diff) of <0.1 indicated an adequate balance between the two groups.

**Table 2:** Hazard ratios of the primary and secondary outcomes between the malnutrition group and control group.

Outcomes	Patients with Outcome		Hazard Ratio (95% CI)	*p* Value
	Malnutrition Group	Control Group		
Primary outcome				
Post-COVID-19 condition or all-cause hospitalization, or death	2683	1383	2.315 (2.170 to 2.471)	<0.0001
Secondary outcomes				
All-cause hospitalization	2283	1257	2.151 (2.008 to 2.304)	<0.0001
All-cause mortality	542	138	4.459 (3.699 to 5.376)	<0.0001
Post-COVID-19 condition	109	45	2.707 (1.913 to 3.832)	<0.0001

CI: confidence interval.

**Table 3:** Subgroup analyses of the primary outcomes between the malnutrition and control groups.

Subgroup Analyses of Primary Outcome	Patients with Outcome		Hazard Ratio (95% CI)	*p* Value
	Malnutrition Group	Control Group		
Gender				
Male	1398	727	2.306 (2.108 to 2.523)	<0.0001
Female	1260	687	2.203 (2.007 to 2.417)	<0.0001
Age				
18–64 y/o	1122	459	2.848 (2.555 to 3.175)	<0.0001
≥65 y/o	1649	1053	1.897 (1.756 to 2.050)	<0.0001
Race				
White	2044	1039	2.337 (2.169 to 2.518)	<0.0001
American Indian or Alaska Native	10	10	4.777 (1.032 to 22.113)	0.0271
Native Hawaiian or Other Pacific Islander	10	10	1.596 (0.568 to 4.489)	0.3698
Black or African American	485	262	2.163 (1.860 to 2.514)	<0.0001
Asian	108	49	2.863 (2.042 to 4.013)	<0.0001
Vaccination status				
Unvaccinated	2055	1088	2.261 (2.101 to 2.433)	<0.0001
Receiving ≥ 2 doses vaccination	202	109	2.085 (1.652 to 2.632)	<0.0001
Antiviral agent status				
Receiving antiviral agent	50	23	2.736 (1.669 to 4.486)	<0.0001
Not receiving antiviral agent	2920	1540	2.246 (2.112 to 2.390)	<0.0001

CI: confidence interval; y/o: years old.

**Table 4:** Subgroup analysis of the secondary outcomes between malnutrition and control groups.

Subgroups	All-Cause Hospitalization		All-Cause Mortality		Post-COVID-19 Condition	
	HR (95% CI)	*p* Value	HR (95% CI)	*p* Value	HR (95% CI)	*p* Value
Gender						
Male	2.133 (1.940 to 2.344)	<0.0001	5.022 (3.828 to 6.587)	<0.0001	3.669 (2.164 to 6.219)	<0.0001
Female	2.013 (1.825 to 2.220)	<0.0001	5.143 (3.840 to 6.888)	<0.0001	2.261 (1.339 to 3.818)	0.0017
Age						
18–64 y/o	2.634 (2.351 to 2.950)	<0.0001	7.397 (4.861 to 11.256)	<0.0001	3.949 (2.345 to 6.651)	<0.0001
≥65 y/o	1.718 (1.583 to 1.866)	<0.0001	3.801 (3.100 to 4.660)	<0.0001	1.873 (1.160 to 3.025)	0.0091
Race						
White	2.118 (1.956 to 2.293)	<0.0001	5.024 (4.045 to 6.241)	<0.0001	2.337 (1.646 to 3.32)	<0.0001
American Indian or Alaska Native	4.255 (0.903,20.038)	0.0460	1.010 (0.063 to 16.144)	0.9945	-	-
Native Hawaiian or Other Pacific Islander	1.246 (0.418 to 3.712)	0.6920	-	-	-	-
Black or African American	2.101 (1.796 to 2.458)	<0.0001	2.852 (1.843 to 4.413)	<0.0001	2.545 (1.078 to 6.624)	0.0472
Asian	2.762 (1.938 to 3.936)	<0.0001	7.821 (2.314 to 26.429)	<0.0001	2.347 (0.430 to 12.828)	0.3102
Vaccination status						
Unvaccinated	2.128 (1.969 to 2.301)	<0.0001	3.563 (2.913 to 4.358)	<0.0001	3.164 (2.113 to 4.736)	<0.0001
Receiving ≥ 2 doses vaccination	1.946 (1.514 to 2.500)	<0.0001	3.401 (1.939 to 5.963)	<0.0001	1.927 (0.712 to 5.210)	0.1884
Antiviral agent status						
Receiving antiviral agent	2.641 (1.588 to 4.393)	<0.0001	11.964 (1.531 to 93.479)	0.0025	1.134 (0.071 to 18.132)	0.9290
Not receiving antiviral agent	2.068 (1.937 to 2.208)	<0.0001	4.158 (3.492 to 4.952)	<0.0001	3.234 (2.296 to 4.556)	<0.0001

CI: confidence interval, HR: hazard ratio, y/o: years.

## Data Availability

The data presented in this study are available on request from the corresponding author.
